# Home-Based Orolingual Exercise Improves the Coordination of Swallowing and Respiration in Early Parkinson Disease: A Quasi-Experimental Before-and-After Exercise Program Study

**DOI:** 10.3389/fneur.2018.00624

**Published:** 2018-07-30

**Authors:** Chin-Man Wang, Wann-Yun Shieh, Chan-Shien Ho, Yu-Wei Hu, Yih-Ru Wu

**Affiliations:** ^1^Department of Physical Medicine and Rehabilitation, Chang Gung Memorial Hospital, Taipei, Taiwan; ^2^College of Medicine, Chang Gung University, Taoyuan, Taiwan; ^3^Department of Computer Science and Information Engineering, Chang Gung University, Taoyuan, Taiwan

**Keywords:** Parkinson disease, orolingual exercise, home-based program, swallowing and respiration coordination, dysphagia, noninvasive assessment

## Abstract

**Introduction:** The coordination of swallowing and respiration is important for safety swallowing without aspiration. This coordination was affected in Parkinson disease (PD). A noninvasive assessment tool was used to investigate the effect of an easy-to-perform and device-free home-based orolingual exercise (OLE) program on swallowing and respiration coordination in patients with early-stage PD.

**Materials and Methods:** This study had a quasi-experimental before-and-after exercise program design. Twenty six patients with early-stage PD who were aged 62.12 ± 8.52 years completed a 12-week home-based OLE program. A noninvasive assessment tool was used to evaluate swallowing and respiration. For each patient, we recorded and analyzed 15 swallows (3 repeats of 5 water boluses: 1, 3, 5, 10, and 20 mL) before and after the home-based OLE program. Oropharyngeal swallowing and its coordination with respiration were the outcome measures. The frequency of piecemeal deglutition, pre- and post-swallowing respiratory phase patterns, and parameters of oropharyngeal swallowing and respiratory signals (swallowing respiratory pause [SRP], onset latency [OL], total excursion time [TET], excursion time [ET], second deflexion, amplitude, and duration of submental sEMG activity, and amplitude of laryngeal excursion) were examined.

**Results:** The rate of piecemeal deglutition decreased significantly when swallowing 10- and 20-mL water boluses after the program. In the 1-mL water bolus swallowing trial, the rate of protective pre- and post-swallowing respiratory phase patterns was significantly higher after the program. For the parameters of oropharyngeal swallowing and respiratory signals, only the amplitude of laryngeal excursion was significantly lower after the program. Moreover, the volume of the water bolus significantly affected the SRP and duration of submental sEMG when patients swallowed three small water bolus volumes (1, 3, and 5 mL).

**Conclusion:** The home-based OLE program improved swallowing and its coordination with respiration in patients with early-stage PD, as revealed using a noninvasive method. This OLE program can serve as a home-based program to improve swallowing and respiration coordination in patients with early-stage PD.

## Introduction

The incidence of oropharyngeal dysphagia (OD) was reported to be high in patients with Parkinson disease (PD) (1). Subclinical dysphagia has been reported to appear in early-stage PD (2). Moreover, swallowing function deterioration with the progression of PD is common. Dysphagia may be associated with choking, dehydration, malnutrition, aspiration pneumonia, and poor quality of life (3), and severe dysphagia may cause mortality (4). Therefore, to prevent or treat swallowing function deterioration, implementing an easy-to-perform home-based program is crucial in early-stage PD (5).

Rehabilitation swallowing therapy (RST) includes indirect and direct swallowing training. Indirect swallowing training without oral feeding is safer than direct swallowing training involving oral feeding, which requires supervision under a therapist in the beginning. Indirect swallowing training includes thermal stimulation, oral motor exercises, and lingual exercises. Exercises are focused on strengthening the tongue muscles, submental muscles, and suprahyoid muscles. Isometric tongue-strengthening exercises have been reported to improve swallowing function in elderly people and patients with stroke, and performing a sensorimotor task has been suggested for strengthening specific muscles (6, 7). However, a feedback mechanism is required for patients performing muscle-strengthening exercises, which is not convenient for a home-based training program. Other treatment programs requiring devices for sEMG feedback (8), neuromuscular electrical stimulation (9, 10), and expiratory muscle-strengthening therapy (EMST) (11, 12) are not easy to perform and unsuitable for home-based programs, which are performed without supervision by a therapist. The development of a device-free and easy-to-perform home-based program should be prioritized for patients with early-stage PD.

Tongue movements are essential for safe swallowing and effective swallowing and respiration coordination. Tongue movement is controlled by the hypoglossal nerve, and the hypoglossal nucleus is located in the caudal pons and ventral medulla (13), which are located near the swallowing and respiration central pattern generators in the brain stem (14). A previous study demonstrated that lingual paralysis impaired swallowing and its coordination with breathing in rats (15). In patients with acute stroke, pneumonia is frequently associated with tongue movement deficits (16). Studies have demonstrated the positive effect of orolingual exercise (OLE) on dysphagia in patients with stroke and neurogenic disorders (7, 17–19). However, few studies have examined the effectiveness of OLE in patients with PD (20, 21). A home-based OLE program does not require supervision by the clinical care team and only requires education impartment to patients. Therefore, “safety” and “easy execution” are the major components in the design of a home-based OLE program. Our OLE home program included effortful dry (saliva) swallowing and lingual exercises, which are safe, easy, and device-free exercises.

Previous studies have reported swallowing and respiratory dysfunction in PD (22–25), but no longitudinal follow-up study has evaluated the therapeutic effect of RST on swallowing function and respiration coordination in PD. We hypothesized that a home-based OLE program including effortful dry (saliva) swallowing and lingual exercises can improve the coordination of swallowing and respiration. Thus, this study was conducted to investigate the effectiveness of a home-based OLE program on the outcomes of swallowing and respiration coordination by using an objective noninvasive tool.

## Material and methods

### Ethics statement

Ethics approval was granted by the Institutional Review Board of our hospital (no. 102-5410B). Each participant was able to understand verbal instructions and signed informed consent prior to participation.

### Participants

In the home-based OLE program, we recruited 26 patients with PD who were referred from the department of neurology. All of these patients completed the training program and follow-up noninvasive swallowing and respiration examinations after undergoing 12 weeks of the exercise program. The enrolled patients had stage I–III PD according to the Hoehn and Yahr disability scale (Table 1A). All patients were also evaluated using the Unified Parkinson Disorder Rating Scale (UPDRS), with its subscales including speech, salivation, and swallowing (Table 1B). This study recruited PD patients with subtle symptoms of subclinical dysphagia and those with poor self-perceived dysphagia. We also recruited patients with early-stage PD who had no self-perceived salivation and swallowing disorders. This study was conducted from August 2014 to August 2015.

**Table 1A:** Characteristics of patients with PD.

	***n* = 26**
Age (years)	62.12 ± 8.52
BMI (kg/m^2^)	22.26 ± 2.47
Gender (M/F)	19/7
Duration of disease (years) (1~19)	8.5 ± 4.65
H&Y (1–3)	1.84 ± 0.811
MMSE (24–30)	28.62 ± 1.472

**Table 1B:** Subscales of the UPDRS in patients with PD (*n* = 26).

**Scale**	**Speech (%)**	**Salivation (%)**	**Swallowing (%)**
0	0 (0)	8 (30.8)	11 (42.3)
1	24 (92.3)	15 (57.7)	7 (26.9)
2	2 (7.7)	3 (11.5)	7 (26.9)
3	0 (0)	0 (0)	1 (3.8)
4	0 (0)	0 (0)	0 (0)

*PD, Parkinson disease; BMI, body mass index; M, male; F, female; H&Y, hoehn & yahr disability scale; MMSE, mini-mental state examination; UPDRS, unified parkinson disorder rating scale*.

### Home-based OLE protocol

**Figure 1:**
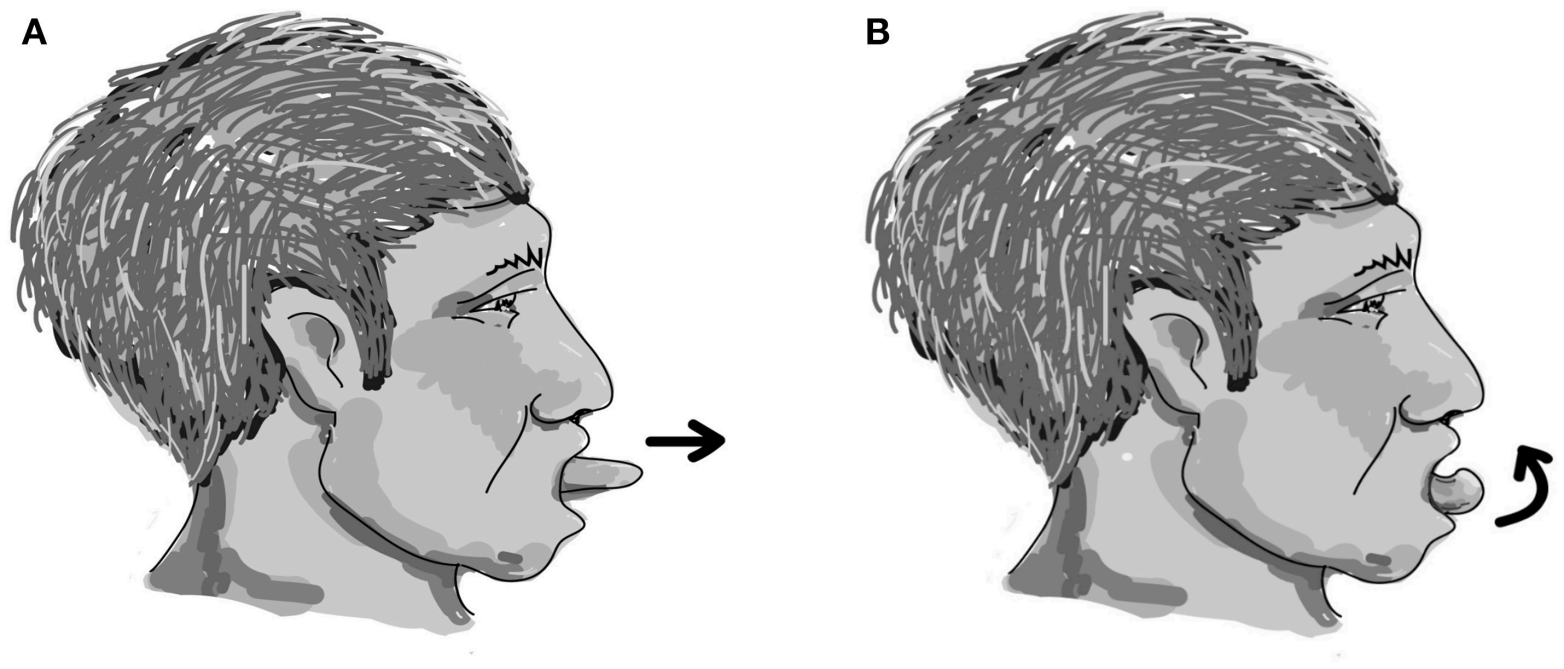
**(A)** Tongue protruding out. **(B)** Tongue rolling back.

The easy-to-perform and device-free home-based OLE program was designed with each cycle including two repetitions of effortful dry (saliva) swallowing, two repetitions of tongue protruding out (Figure 1A), followed by two repetitions of tongue rolling back (Figure 1B). One session included 25 cycles, and two sessions were performed each day for 5 days a week for a period of 12 weeks. The effectiveness of the 12-week home-based OLE program including lingual exercises and effortful dry (saliva) swallowing was evaluated.

### Hardware, software, and protocol

Similar to our previous studies (25–28), we used an electrophysiological monitoring system (Biopac MP 100 system) and AcqKnowledge software for data recording and analysis. By using the Biopac 100 system, we simultaneously recorded swallowing and respiration biosignals, including submental surface electromyography (sEMG), nasal airflow, and thyroid cartilage excursion, during swallowing. For each patient, we recorded and analyzed 15 swallows (3 repeats of the following 5 water boluses: 1, 3, 5, 10, and 20 mL) before and after the 12-week home-based OLE program.

### Outcome measures

Noninvasive assessment of swallowing and respiration coordination was conducted at two time points: at the baseline (before the program) and after the program.

### Definition of respiration coordination and oropharyngeal swallowing signals

*1. Piecemeal deglutition (**29**)*: Multiple swallows are observed when the bolus volume fed in the mouth exceeds the dysphagia limit.
*2. Pre- and post-swallowing respiratory phase patterns (**30**)*: The expiration–expiration (Ex–Ex) pre- and post-swallowing respiratory phase pattern is the major and protective swallowing respiratory phase pattern.
*3. Parameters (latency, duration, and amplitude) of oropharyngeal swallowing and respiratory signals*: These parameters included swallowing respiratory pause (SRP), onset latency (OL), total excursion time (TET), excursion time (ET), second deflexion of laryngeal excursion, amplitude and duration of submental sEMG activity, and amplitude of laryngeal excursion.

All the aforementioned parameters have been defined and used to analyze swallowing and its coordination with respiration in our previous studies (25–28).

### Statistical analysis

SPSS software (version 12.0; SPSS Inc., Chicago, IL) was used for all statistical analyses. For each patient, three swallowing trials of each bolus volume were conducted, with a total of 15 swallows. The chi-square test or Fisher exact test was used to analyze patient data on piecemeal deglutition (10 and 20 mL) and pre- and post-swallowing respiratory phase patterns (without piecemeal deglutition), which are expressed as numbers and frequencies. Oropharyngeal and respiratory signals alone (without piecemeal deglutition) were averaged and analyzed. Repeated-measures analysis of variance was used to examine the interaction and main effect. Independent variables were SRP and oropharyngeal parameters, and dependent variables were measurements obtained before and after exercises and three small bolus volumes (1, 3, and 5 mL). The level of α was set at 0.05. A *P*-value < 0.05 was considered statistically significant. Using G^*^Power 3, we calculated a sample size of more than 27 for repeated measures ANOVA to detect large effects (0.27) (31), with 80% power at the significance level of 0.05.

## Results

### Patient characteristics

Tables 1A,B list the characteristics of patients with PD who completed follow-up (*n* = 26).

### Therapeutic effect of the 12-weeks home-based OLE program

#### Piecemeal deglutition

Compared with the baseline, the probability of piecemeal deglutition decreased significantly after the program in patients with PD in both 10- and 20-mL water bolus swallowing trials (*P* < 0.001 and *P* = 0.001, respectively; Table 2A).

**Table 2A:** Piecemeal swallowing of 10- and 20-mL water boluses before and after OLE program.

**Water bolus**		**Piecemeal (no) (%)**	**Piecemeal (yes) (%)**	***P-*value**
10 mL	Before program	21 (27)	56 (73)	< 0.001
(*n* = 26 × 3–1)	After program	47 (61)	30 (39)	
20 mL	Before program	9 (12)	69 (88)	0.001
(*n* = 26 × 3)	After program	27 (35)	51 (65)	

### Pre- and post-swallowing respiratory phase patterns

The rate of protective (Ex–Ex) respiratory phase patterns tended to increase after the program in swallowing trials of the five water bolus volumes without piecemeal deglutition. However, a significant difference was observed before and after the program in only the 1-mL water bolus swallowing trial without piecemeal deglutition (*P* = 0.045; Table 2B).

**Table 2B:** Pre- and post-swallowing respiratory phase pattern.

**Water bolus (mL)**		**Ex-Ex (%)**	**Non Ex-Ex (%)**	***P-*value**
1	Before program	10 (22)	35 (78)	0.042^*^
	After program	25 (41)	36 (59)	
3	Before program	11 (29)	27 (71)	0.410
	After program	24 (37)	41 (63)	
5	Before program	13 (33)	26 (67)	0.148
	After program	27 (48)	29 (52)	
10	Before program	8 (38)	13 (62)	0.612
	After program	21 (45)	26 (55)	
20	Before program	2 (22)	7 (78)	0.531
	After program	9 (33)	18 (67)	

*No. of swallows in each cell is 78 (26 × 3) in Table, but only swallows without PMD were analyzed. PMD, piecemeal deglutition; Ex–Ex, expiratory–expiratory; OLE, orolingual exercise*.

* *P < 0.05*.

### Parameters (latency, duration, and amplitude) of oropharyngeal swallowing and respiratory signals (during swallowing trials of three small water boluses: 1, 3, and 5 mL)

**Figure 2:**
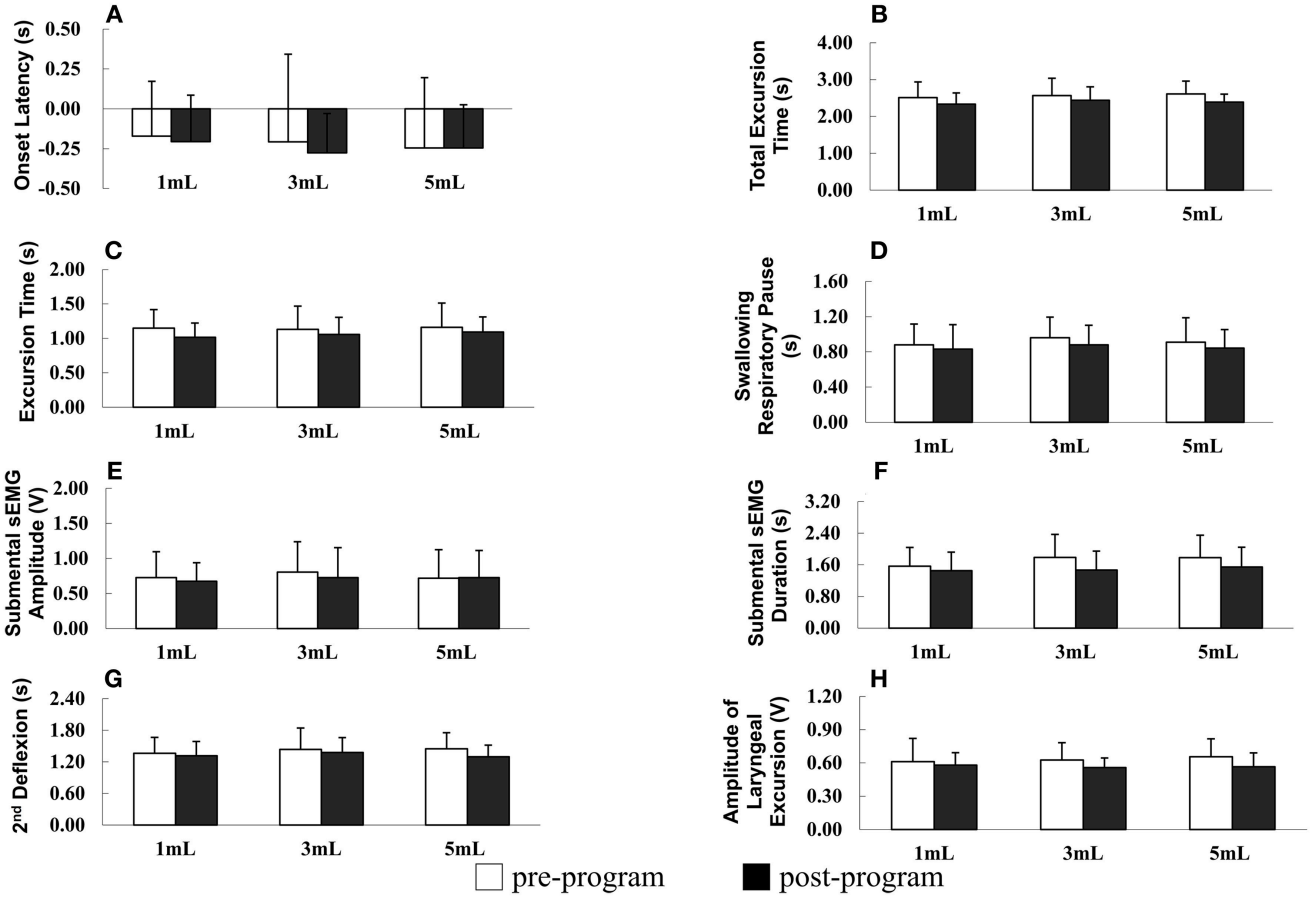
**(A–H)** Parameters of oropharyngeal swallowing and respiratory signals before and after the home-based orolingual exercise program in patients with early-stage Parkinson disease. s, seconds; sEMG, surface electromyography; V, voltage; 2^nd^: second.

The results of repeated measures ANOVA revealed no significant difference in SRP before and after the program in patients with PD (*F* = 1.895, *P* = 0.178), indicating that SRP was not longer after the program in patients with PD. However, SPR significantly differed among the different water bolus volumes (*F* = 3.621, *P* = 0.032; Figure 2D).

OL did not differ significantly before and after the program (*F* = 0.381, *P* = 0.541) and among the three small bolus volumes (*F* = 1.167, *P* = 0.313; Figure 2A). In addition, in patients with PD, the TET did not differ significantly before and after the program (*F* = 2.588, *P* = 0.117) and among the three small bolus volumes (*F* = 0.364, *P* = 0.696; Figure 2B). No significant difference was observed in the ET before and after the program in patients with early-stage PD (*F* = 0.137, *P* = 0.868), and the ET did not differ significantly among the three small bolus volumes (*F* = 2.176, *P* = 0.149; Figure 2C). The duration of the second deflexion did not differ significantly before and after the program (*F* = 0.668, *P* = 0.420). Moreover, no significant difference was observed in the second deflexion among the three small bolus volumes (*F* = 1.054, *P* = 0.351; Figure 2G). The amplitude of submental sEMG was not significantly different before and after the program in patients (*F* = 2.022, *P* = 0.165). The water bolus volume significantly affected the parameters when patients swallowed the three small bolus volumes (*F* = 3.717, *P* = 0.030; Figure 2E). The amplitude of submental sEMG decreased as the bolus volume increased from 1 to 5 mL. For submental sEMG activity, the contraction duration did not differ significantly before and after the program (*F* = 2.807, *P* = 0.103) as well as among the three small bolus volumes (*F* = 1.099, *P* = 0.399; Figure 2F). The amplitude of laryngeal excursion differed significantly before and after the program (*F* = 4.634, *P* = 0.039), but it did not differ significantly among the three small bolus volumes (*F* = 0.637, *P* = 0.527; Figure 2H). No significant interactions were found in any of the measured parameters between groups and boluses.

In summary, a low rate of piecemeal deglutition (10 and 20 mL), a high rate of protective respiratory phase patterns, and a low amplitude of laryngeal excursion were observed after the 12-week home-based OLE program. Regarding the temporal parameters of oropharyngeal swallowing and respiratory signals, the duration of the SRP, TET, ET, and second deflexion associated with swallowing the three small water bolus volumes (1, 3, and 5 mL) tended to be shorter after the OLE program than before the program in patients with PD; however, this difference was not statistically significant. Taken together, our data derived from the noninvasive swallowing and respiration assessment indicate that the coordination of swallowing and respiration in patients with early-stage PD improved after the 12-week home-based OLE program.

## Discussions

According to our review of the literature, this is the first study to evaluate the effects of an easy-to-perform and device-free home-based OLE program on swallowing and respiration coordination by using a noninvasive examination. Our data demonstrate this home-based OLE program including effortful dry (saliva) swallowing and lingual exercises exerted a positive effect on swallowing and respiration coordination in patients with early-stage PD.

Swallowing is a consequence of movement patterns. Accordingly, task-specific exercises for swallowing training are based on the effectiveness of skill training in the limbs (32–34). Task-specific exercises emphasize repeated practices for skill enhancement. Thus, after repeated practices of effortful saliva swallowing, the optimal swallowing ability may accentuate through the recruitment of more motor units (35). Oral motor training by tongue-task training through the central neural level of the corticobulbar pathway improved the motor control of the tongue (36, 37). Effortful swallowing increased the oropharyngeal pressure and improved the biomechanical and bolus flow aspects of swallowing (38). In addition, during saliva and liquid swallowing, dynamic lingual—mandibular coupling movements were demonstrated (39, 40). Resistance strengthening exercise of the opening of the jaw has been reported to improve the opening of the upper esophageal sphincter (41, 42). The findings of previous studies on task-specific exercises and jaw opening strengthening exercises might explain the observed positive effect of our home-based OLE program including effortful dry (saliva) swallowing and lingual exercises with the tongue protruding out that also simultaneously combined jaw opening without resistance.

Patients with PD have poor oral control (43) and a high piecemeal deglutition rate (24). Piecemeal deglutition may be due to poor oral motor control and/or oropharyngeal dysfunction, and although it is a phenomenon for safe swallowing, it reduces the efficiency of swallowing. In our study, a decreased rate of piecemeal deglutition was observed in 10- and 20-mL water bolus swallowing trials after the 12-week home-based OLE program. This finding indicates that the efficiency of swallowing improved after the home-based OLE program in our patients with early-stage PD. In addition, the probability of the pre- and post-swallowing respiratory phase pattern of Ex–Ex increased after the exercise program, suggesting the occurrence of a more protective swallowing respiratory phase pattern after the program.

For clinical application, this home-based OLE program may be useful in early-stage PD and can be used as a regular home exercise program. An early intervention to prevent the deterioration of oral motor and swallowing function is important. Designing an easy, simple, and device-free home-based program is the most suitable option. Future studies with similar designed investigations and programs will be important for patients with later stages of PD. The therapeutic effects of other oral motor training programs and respiratory muscle-strengthening exercises are also crucial in early PD.

## Limitations

Our study has some limitations. First, this study did not apply a randomized design. Because the variable clinical symptoms and signs and heterogeneous courses in PD, performing a randomized study is difficult. However, PD is a chronic progressive disease, and spontaneous recovery is not expected with time. Second, our patients were in the stationary status receiving an unaltered protocol of anti-PD medication for more than 2 months and were regularly followed up at the outpatient clinics of the neurological department. Thus, our conclusion may not be generalizable. Third, we did not recruit patients with late-stage PD or those with severe dysphagia requiring tube feeding; thus, the results of this study cannot be generalized to such patients. Finally, no long-term follow-up was conducted after the training program in our study; thus, the long-standing effects of the training program could not be investigated.

## Conclusions

The easy-to-perform and device-free home-based OLE program exerted a positive effect on oropharyngeal swallowing and respiration coordination in patients with early-stage PD. This OLE program can be useful in clinical application for dysphagia care in early PD.

## Author contributions

C-MW, Y-WH, W-YS, and C-SH performed the research. C-MW and Y-RW designed the research study. C-MW and W-YS contributed essential reagents or tools. C-MW and W-YS analyzed the data. C-MW and Y-RW wrote the paper.

### Conflict of interest statement

The authors declare that the research was conducted in the absence of any commercial or financial relationships that could be construed as a potential conflict of interest.
